# A Novel Energy Recovery System for Parallel Hybrid Hydraulic Excavator

**DOI:** 10.1155/2014/184909

**Published:** 2014-10-22

**Authors:** Wei Li, Baoyu Cao, Zhencai Zhu, Guoan Chen

**Affiliations:** ^1^School of Mechanical Engineering, China University of Mining and Technology, Xuzhou 221116, China; ^2^Shanghai Chuangli Group Co., Ltd., Shanghai 201706, China

## Abstract

Hydraulic excavator energy saving is important to relieve source shortage and protect environment. This paper mainly discusses the energy saving for the hybrid hydraulic excavator. By analyzing the excess energy of three hydraulic cylinders in the conventional hydraulic excavator, a new boom potential energy recovery system is proposed. The mathematical models of the main components including boom cylinder, hydraulic motor, and hydraulic accumulator are built. The natural frequency of the proposed energy recovery system is calculated based on the mathematical models. Meanwhile, the simulation models of the proposed system and a conventional energy recovery system are built by AMESim software. The results show that the proposed system is more effective than the conventional energy saving system. At last, the main components of the proposed energy recovery system including accumulator and hydraulic motor are analyzed for improving the energy recovery efficiency. The measures to improve the energy recovery efficiency of the proposed system are presented.

## 1. Introduction

At present, with the development of world economic construction, the cost of the energy has increased rapidly. Pollution and global warming have become extremely serious problems that the world has to face. As the most typical equipment of engineering machinery industry, the hydraulic excavator plays an important role in construction, water conservancy, railway, and highway. However, due to the complex working condition and frequent load changing, only 20% of the engine output power is utilized in a conventional type excavator [1]. Combined controls of actuators require distribution of flows and interflows, which increase loop loss. Meanwhile, the potential of working device, the kinetic energy of the turning body, and braking bodywork are converted into heat in the main throttle valve. This will lead to energy waste and temperature rising of system. The hydraulic component of system is damaged after the long time working. Therefore the energy recovery of working device has an important significance for improving energy utilization ratio for the conventional hydraulic excavator [2, 3].

Several researches on the energy saving of the conventional hydraulic excavator have been proposed [4–6]. In general, the approaches for improving energy utilization ratio can be categorized into two types. One is improving the efficiencies of individual hydraulic components and the other is developing efficient hydraulic systems [7]. Furthermore, the most effective ways to create more efficient systems are matching the output power of pumps to the desired power of loads and regenerating the recoverable energy of actuators such as braking kinetic energy or gravitational potential energy.

Hybrid is a new power system which is widely used in automotive industry [8–10]. It can be assigned to either series hybrid, parallel hybrid, or their combination. The hybrid electric vehicle (HEV) utilizes more than two different motive powers to propel the wheel, one of which is electric energy. The hybrid system can thoroughly optimize the two energy configuration and take advantage of the benefits provided by them [11]. Therefore, compared with the traditional vehicle, HEV not only has a potential to improve the fuel efficiency, but also reduce the emission.

Based on the successful application of the hybrid system in automotive industry, it attracts a lot of the world's largest companies and institute's interest. Many researches on the application of the hybrid technology in hydraulic excavator have been done. In order to enhance fuel economy of hybrid excavator system, Gong et al. [12] introduce a control strategy based on equivalent fuel consumption. The results show that the control strategy can effectively optimize the hybrid power distribution and improve fuel economy. Liu et al. [13] find a versatile method designing the parameters of main components of hydraulic excavator. The method has simple calculation process and it can be used to carry out parameter matching on different hybrid system. Lin et al. [14] deal with the method of how to regenerate the potential energy for a hybrid hydraulic excavator. The simulation results show that it is possible to increase the efficiency of the generator and downsize the generator by adding the hydraulic accumulator to the system.

This paper mainly presents a new hybrid hydraulic excavator energy recovery system which combines the hydraulic accumulator and the electric regeneration unit together. In this system, the accumulator and the regeneration unit are installed in the return oil lines. In some operating conditions, the excess energy supplied by the pump can be converted to electricity and stored in the battery. The cylinder velocities are governed by the displacement of hydraulic motor. The proposed system is simulated by AMESim software. The energy recovery efficiency of the proposed system is clearly verified through simulation results in comparison with the conventional energy recovery system. At last, in order to improve the energy recovery efficiency of the proposed system, the main components of the proposed energy recovery system including accumulator and hydraulic motor are analyzed. The results show that the different key parameters of components have a great influence on the energy recovery efficiency.

## 2. Design of the System Scheme

### 2.1. Energy Consumption Analysis of the Traditional Excavator

**Figure 1:**
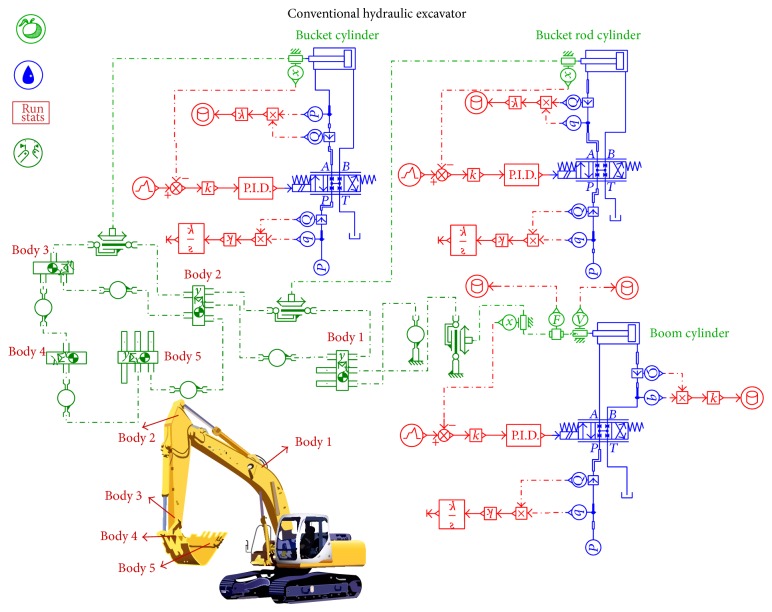
AMESim model of conventional hydraulic excavator.

As the hydraulic excavator starts to work, the boom cylinder piston can expand and contract twice during a work period as well as the bucket cylinder and the bucket rod cylinder. Due to the high frequency of use, three cylinders mentioned above are analyzed based on 25*t* hydraulic excavator (middle type hydraulic excavator). Simulation with conventional hydraulic excavator has been carried out by using AMESim software. The model built in AMESim is shown in Figure 1. In order to simplify the model, there are two assumptions for the system.

1. The pumps in the hydraulic excavator system are replaced by three constant pressure sources, the input pressure *p*_in_ = 200 bar, and they supply the flow rate which the actuators need.
2. There is not any energy loss in the hydraulic circuit and components but for the electrohydraulic directional control valves, and the pressure drop *p*_drop_ = 20 bar.

Run the simulation for a whole work period of the hydraulic excavator. The power of the pumps *P*_in_ and the power of the cylinders *P*_out_ are calculated by the following, respectively,

(1) $$\begin{array}{c} P_{\text{in}}=p_{\text{in}}\cdot q_{\text{in}}, \end{array}$$

(2) $$\begin{array}{c} P_{\text{out}}=p_{\text{out}}\cdot q_{\text{out}}, \end{array}$$

where *p*_in_ and *q*_in_ are the pressure and flow rate of the pumps and *p*_out_ and *q*_out_ are the pressure and flow rate of the cylinders.

**Figure 2:**
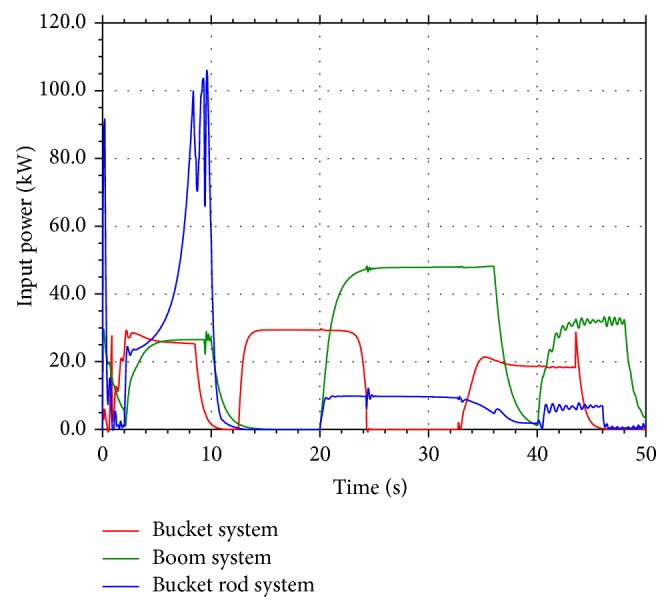
Input power of the three cylinder systems.

**Figure 3:**
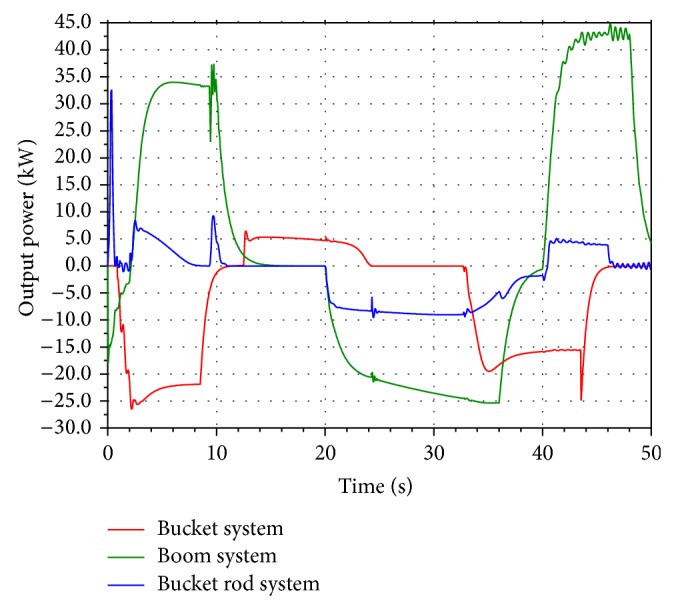
Output power in the return oil lines.

According to the operating condition of the hydraulic excavator, when the boom cylinder piston is contracting and the bucket cylinder and the bucket rod cylinder piston are expanding, the excess potential energy in the return oil lines can be recycled and reused. The input power of the three cylinder systems and the output power in the return oil lines are shown in Figures 2 and 3. From Figure 3, it is shown that the part of output power which is greater than zero can be recycled and reused. The input energy and the excess energy which can be recycled of the three cylinder systems are given in Table 1.

From Table 1, it is shown that there is plenty of energy loss in the return oil lines when the boom cylinder piston is contracting. Compared with the bucket rod and bucket systems, the boom system has a high energy recovery percentage. Taking the complexity and cost of the system into consideration, the boom potential energy recovery system in hybrid hydraulic excavator based on an accumulator and a generator is proposed.

### 2.2. Structure of the Boom Potential Energy Recovery System

A new boom potential energy recovery system needs to be designed to satisfy the following requirements:

1. operation of the new boom energy recovery system must be similar to the conventional hydraulic excavator;
2. the new boom energy recovery system must achieve higher working efficiency and save more energy when comparing with the conventional system.

**Figure 4:**
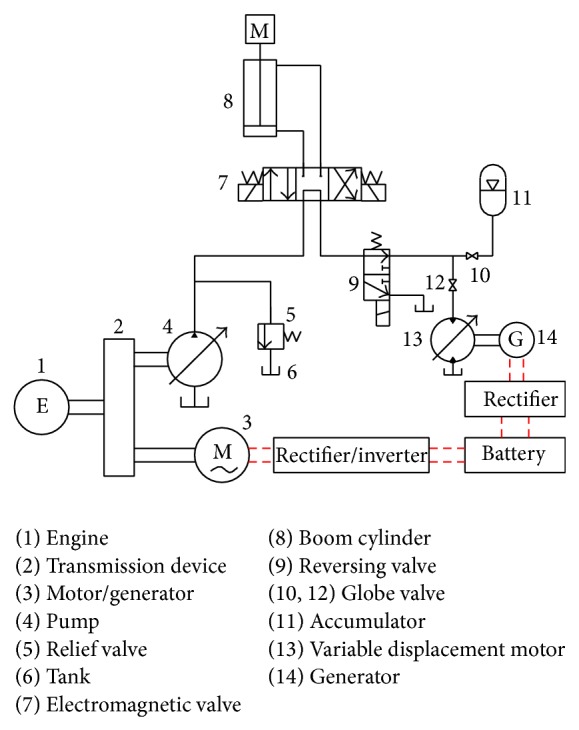
Schematic of proposed boom energy-recovery hydraulic system.

Figure 4 shows the schematic of the proposed hydraulic system. It mainly consists of oil supply system, boom cylinder, control valves, and energy regeneration unit. The pump is driven by the engine and the motor. The pressure oil exporting from the pump was supplied to the boom cylinder system. When the boom cylinder piston is contracting, the excess energy is converted into electrical energy and stored in the battery. Compared with the engine power *P*_*e*_ and the load power *P*_*l*_, there are three kinds of working conditions based on the load change.

1. When *P*_*e*_ > *P*_*l*_, the pump is driven by the engine and the excess power of the engine is converted into electrical energy by the motor and stored in the battery. The motor is working as a generator in this working condition.
2. When *P*_*e*_ < *P*_*l*_, electrical energy stored in the battery is used to drive the motor. The engine and the motor drive the pump together.
3. When the motor power *P*_*e*_ > *P*_*m*_ > *P*_*l*_, the pump is driven by the motor independently and the engine is working in the idle state.

**Figure 5:**
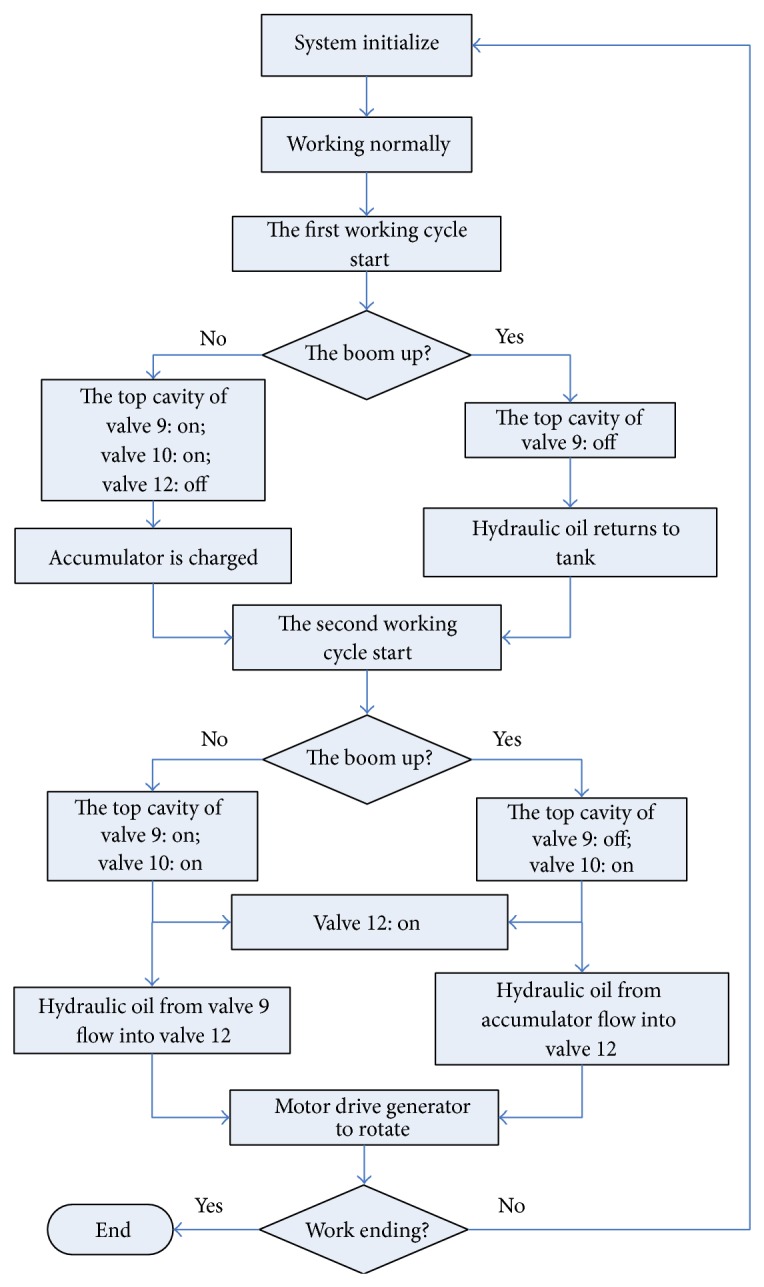
Working flow chart of the energy saving system.

The working flow chart of the proposed system is shown in Figure 5. Compared with the conventional energy recovery system, the pressure oil is charged into the accumulator instead of flowing into the motor directly when the boom arm falls in the first working cycle. Meanwhile, the pressure oil in the accumulator is discharged and flowing into the motor when the boom arm rises in the second working cycle. It makes sure that the generator can rotate continuously in a high speed. Hence, two working cycles of the conventional hydraulic excavator are regarded as a complete working period for the new energy saving system.

## 3. Mathematical Modeling

### 3.1. Boom Cylinder

As shown in Figure 4, the dynamics of the piston of the boom cylinder can be expressed as

(3) $$\begin{array}{c} F_{l}+P_{2}A_{2}-P_{1}A_{1}-b_{c}v_{c}-F_{f}-Mv_{c}^{\prime \prime }=0. \end{array}$$

Continuity equation of hydraulic cylinder piston can be expressed as

(4) $$\begin{array}{c} \frac{Q_{1}}{A_{1}}=\frac{Q_{2}}{A_{2}}, \end{array}$$

where *M* is the equivalent mass of the load, *v*_*c*_ is the velocity of the piston, *F*_*l*_ is the external force, and *P*_1_ and *P*_2_ are the pressures in the large and small chamber of the boom cylinder, respectively. *Q*_1_ and *Q*_2_ are the corresponding flow rate, *A*_1_ and *A*_2_ denote the corresponding working areas, *F*_*c*_ is the coulomb friction force, and *b*_*c*_ is the combined coefficient of damping and viscous friction forces on the load and the rod. The value of *v*_*c*_ is the differential of the piston displacement *x*_*c*_.

### 3.2. Hydraulic Motor

The dynamics of the rotor of the regeneration unit can be expressed as

(5) $$\begin{array}{c} D_{m}\left(P_{3}-P_{4}\right)=J\frac{d\omega_{m}}{dt}+b_{m}\omega_{m}+T_{f}+T_{g}, \end{array}$$

where *ω*_*m*_ is the rotational speed of the hydraulic motor,* J* is the total moment of inertia of the regeneration unit, *T*_*g*_ is the electromagnetic torque of the generator, *T*_*f*_ is the coulomb friction torque, *B*_*m*_ is the combined coefficient of damping and viscous friction torques on the rotor, *D*_*m*_ is the displacement of the hydraulic motor, and *P*_3_ and *P*_4_ are the inlet and outlet pressures of the motor.

Flow continuity equation of the motor can be written as

(6) $$\begin{array}{c} Q_{3}-C_{em}P_{3}-C_{im}\left(P_{3}-P_{4}\right)-D_{m}\omega_{m}=0,\\ D_{m}\omega_{m}+C_{im}\left(P_{3}-P_{4}\right)-C_{em}P_{2}-Q_{4}=0, \end{array}$$

where *C*_*em*_ is the external leakage coefficients of the motor and *C*_*im*_ is the internal leakage coefficient of the motor.

Assuming that there is no loop loss in reversing valve, the flow equation of the chamber between the cylinder and the motor can be written as

(7) A1vc−Cic(P1−P2)−CecP1−Cim(P1−P4)  −ωmDm−Qa=VβedP1dt,

where *C*_*ic*_ is the internal leakage coefficients of the cylinder, *C*_*ec*_ is the external leakage coefficient of the cylinder, *V* is the volume of the hydraulic oil between the boom cylinder and motor, *Q*_*a*_ is the flow of the hydraulic oil stored in the accumulator, and *β*_*e*_ is the volume elastic modulus.

### 3.3. Hydraulic Accumulator

The bladder accumulator is chosen in this boom energy recovery system, according to Boyle law, and the formula is given by

(8) $$\begin{array}{c} p_{0}V_{0}^{n}=p_{1}V_{1}^{n}=p_{2}V_{2}^{n}=p_{a}V_{a}^{n}, \end{array}$$

where *p*_0_, *p*_1_, *p*_2_, *p*_*a*_ denote the initial aeration pressure, initial pressure, terminal state pressure, and free state pressure of accumulator, respectively; *V*_0_, *V*_1_, *V*_2_, *V*_*a*_ are the initial aeration volume, initial volume, terminal state volume, and free state volume of accumulator; *n* is the air polytropic exponent.

*p*_*a*_ and *V*_*a*_ are the random operating state of accumulator; the equality of *p*_0_ *V*_0_^*n*^ = *p*_*a*_ *V*_*a*_^*n*^ is expanded using Taylor expansion; the Taylor expansion is given by

(9) $$\begin{array}{c} \frac{dP_{a}}{dt}=-\frac{nP_{0}}{V_{0}}\frac{dV_{a}}{dt}. \end{array}$$

Flow and air chamber volume of accumulator are *Q*_*a*_ and *V*_*a*_ and the inlet flow rate of accumulator is given by

(10) $$\begin{array}{c} Q_{a}=-\frac{dV_{a}}{dt}. \end{array}$$

Energy equation of accumulator

(11) $$\begin{array}{c} E=-\overset{V_{2}}{\underset{V_{1}}{\int }}{\left(\frac{V_{0}}{V_{a}}\right)}^{n}dV_{a}=\frac{P_{0}V_{0}}{n-1}\left[{\left(\frac{P_{a}}{P_{0}}\right)}^{\left(n-1\right)/n}-1\right]. \end{array}$$

According to the equations above, the flow control system is an obviously nonlinear system. In order to verify its stability and dynamic performance, linearization and Laplace transform are carried out. The transfer function from the hydraulic motor speed to the load force can be expressed as

(12) ωm(s)F(s) =(DmA1Tg)  ×([JM(CnP0+V0)A12nP0Tg+MVA12βe]s2 +[DmM+JA12A12Tg+M(CnP0+V0)nP0A12]s+1)−1,

where *C* is the leakage coefficients of the energy recovery system.

The natural frequency of the proposed energy recovery system can be calculated as

(13) $$\begin{array}{c} \omega_{H}=\sqrt{\frac{1}{JM\left(CnP_{0}+V_{0}\right)/A_{1}^{2}nP_{0}T_{g}+MV/A_{1}^{2}\beta_{e}}}. \end{array}$$

The natural frequency is the lowest frequency of the system. The low natural frequency has an effect on the response speed of the system and energy recovery efficiency. In order to improve the natural frequency and response speed of the system, based on the expression of the natural frequency, the following ways should be taken into consideration.

1. Reducing the loop oil volume *V*: to make the whole system structure compact and high-efficiency, the oil line should be installed effectively and the length of the line should be shortened as soon as possible.
2. Increasing the volume elastic modulus of the hydraulic oil *β*_*e*_: while designing the system and selecting the hydraulic oil, the volume elastic modulus *β*_*e*_ of the oil should have a large value relatively.
3. Reducing the leakage coefficients of the energy recovery system *C*: as it is hardly realistic to eliminate the system leakage, the quality of the hydraulic components chosen in the system should satisfy the long time using performance.
4. Reducing the total moment of inertia of the hydraulic motor *J*: according to the characteristics of the motor, the total moment of inertia decreases along with the decreasing of the displacement, so the displacement of the motor should be reduced to a certain degree. However, the flow rate of the return oil lines will reduce when the displacement of the motor decreases. Hence it has an effect on the working performance of the hydraulic excavator.

## 4. Simulation of the Boom Energy Recovery System

**Figure 6:**
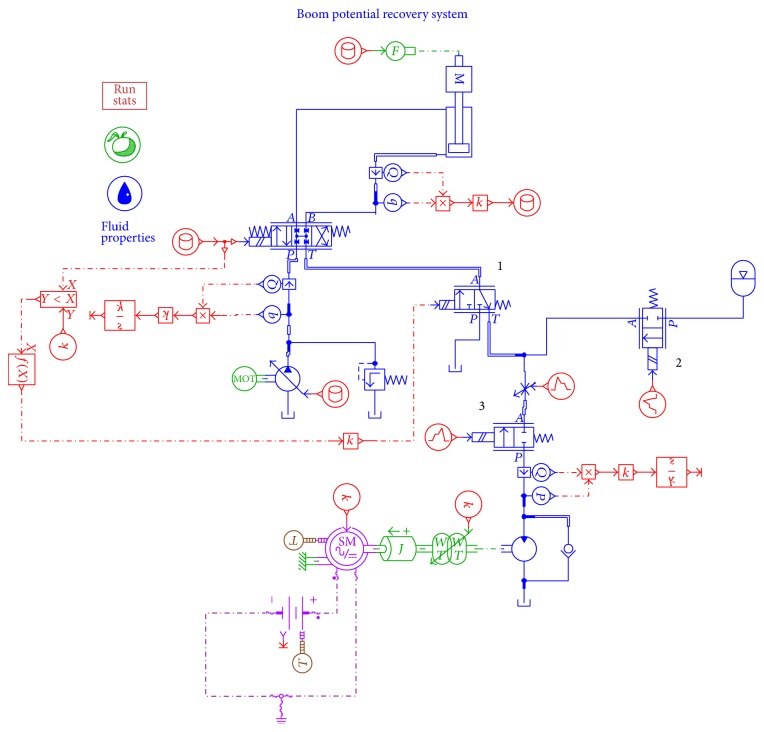
AMESim model of the proposed system.

**Figure 7:**
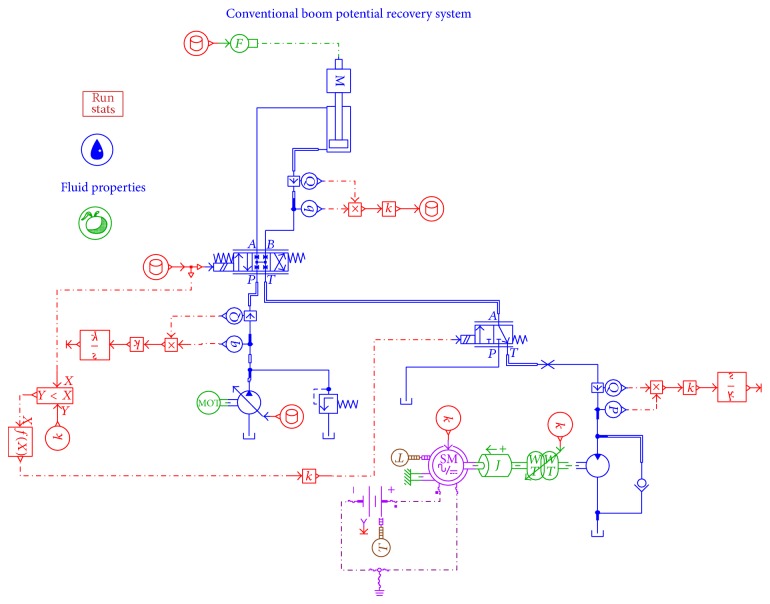
AMESim model of a conventional boom energy recovery system.

In order to verify the energy saving efficiency of the proposed system, simulations with the proposed accumulator-generator system and the conventional energy recovery system have been carried out by using AMESim. It aims to validate the impact of accumulator on energy recovery efficiency.  Figure 6 shows the AMESim model of the proposed system with accumulator, while Figure 7 displays another kind of boom energy recovery system without accumulator. To simplify the system, the engines are replaced by two motors in Figures 6 and 7.

**Figure 8:**
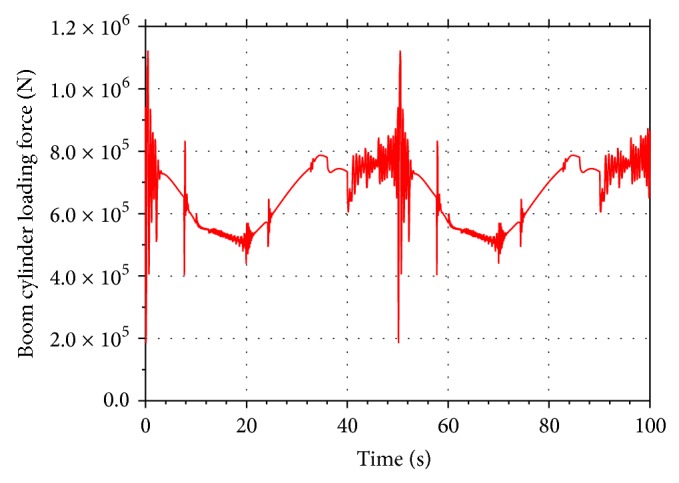
Input load force of the energy saving system.

Including the load force and dimension parameters of the boom cylinder, the setting parameters for the AMESim models are obtained from the conventional hydraulic excavator. The main setting parameters for the two AMESim models are given in Table 2. The input load force of the conventional energy recovery system and the proposed energy recovery system is shown in Figure 8.

**Figure 9:**
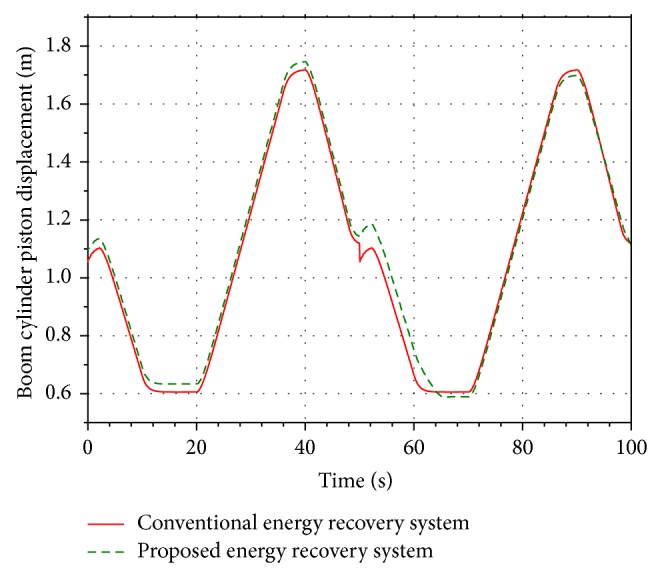
Piston displacement of the boom cylinder.

Run the simulations. The displacement of the boom cylinder in the conventional energy recovery system and the proposed system are shown in Figure 9. Figure 9 shows that the piston displacement of the boom cylinder in the two systems is quite similar. The working performance of the boom cylinder is not affected by the energy recovery system installed in the return oil lines.

**Figure 10:**
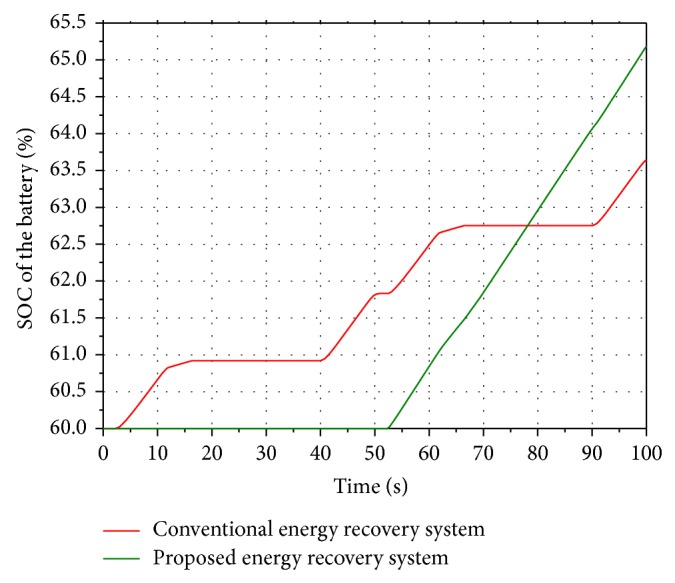
SOC of the battery.

The difference between the SOC (State of Charge) of the batteries in the two systems is shown in Figure 10. For the conventional energy recovery system, the generator starts and stops four times during a working period. According to the mechanical characteristics of the generator, high efficiency depends on high speed and continuous rotation. Because of the accumulator, the generator of the proposed energy recovery system starts and stops only once during a working period. Hence the generator can rotate in a high speed continuously. Compared with the conventional energy saving system, SOC of the battery in the proposed system can rise smoothly. During a whole working period, the value of SOC reaches 65.2%. Finally, the input energy and the energy stored in the batteries of the two energy saving systems are given in Table 3.

Based on Table 3, the value of the energy recovery efficiency in the proposed system is 14.6% while the value of the conventional energy recovery system is 6.6%. It is clear that the proposed boom potential energy recovery system brings higher energy recovery efficiency than the conventional boom potential energy recovery system.

## 5. Analysis of the Main Components in the System

As designing the boom energy recovery system of the hybrid hydraulic excavator, all the components of the system are chosen based on the calculation results and working condition. However, some parameters of the main components have a great influence on the energy recovery efficiency of the proposed system. Inappropriate parameters will lead to the decreasing of the efficiency. Therefore, it is essential to analysis the relationship between the energy recovery efficiency and the key parameters of the main components like the aeration pressure of the accumulator and the displacement of the hydraulic motor. In order to simplify the model, there are three assumptions for the simulation models.

1. Since this study concentrates on the effectiveness of the proposed boom energy recovery system, the working performance of the engine is not taken into consideration. The engine is replaced by an electromotor.
2. The load force and the piston velocity of the boom cylinder in the simulation models are identical with the conventional hydraulic excavator. In other words, the boom cylinder system is working under the same conditions.
3. A generator and a battery are selected as the energy conversion and energy storage units. Regardless of the internal structure of generator and battery, the simulation models are replaced by the universal models.

### 5.1. Analysis of the Accumulator

The key parameters of the accumulator include the aeration pressure and the initial volume. The simulations of the relationship between the parameter values and the system energy recovery efficiency are shown below.

#### 5.1.1. The Aeration Pressure

**Figure 11:**
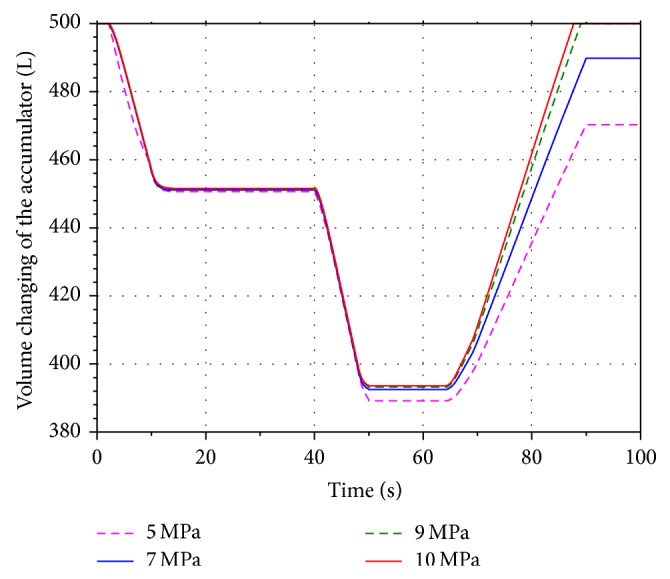
Volume changing of the accumulator.

**Figure 12:**
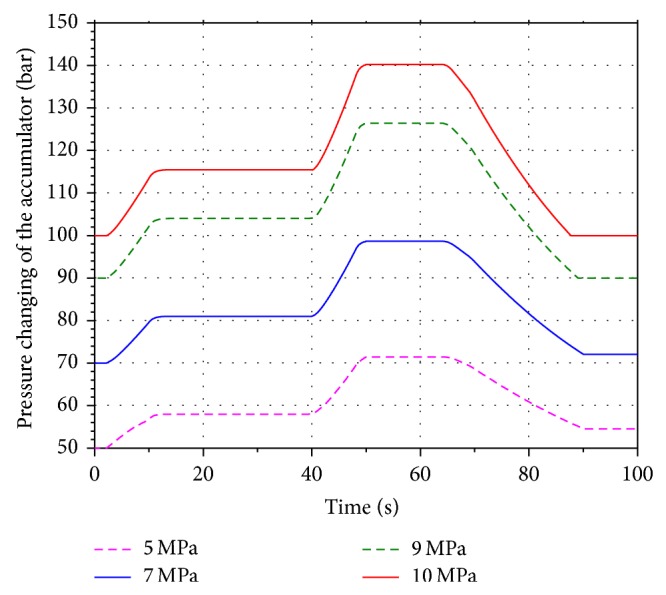
Pressure changing of the accumulator.

**Figure 13:**
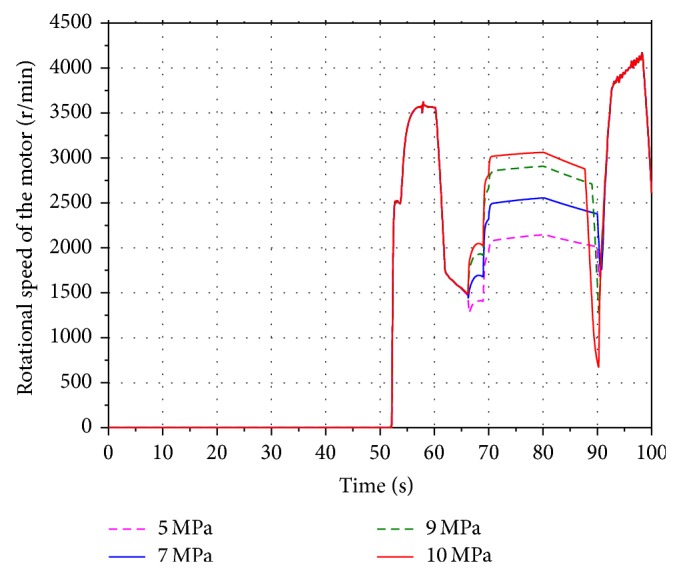
Rotational speed of the hydraulic motor.

**Figure 14:**
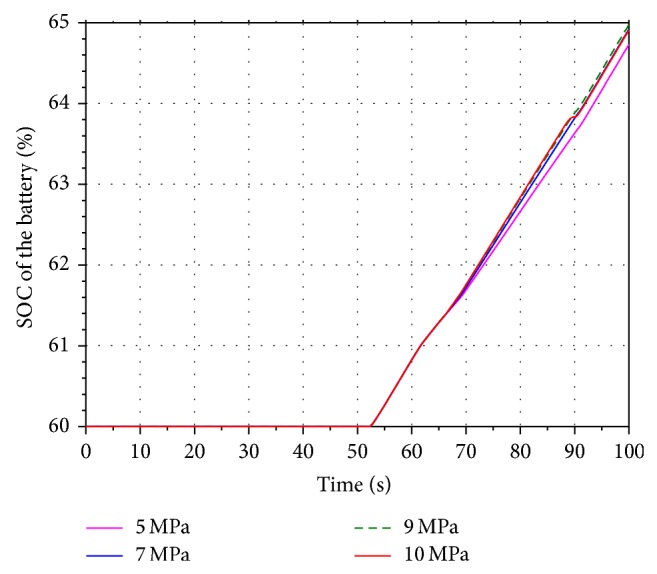
SOC of the battery.

In order to analyze the influence of the different aeration pressure on the energy recovery efficiency, four values 5 MPa, 7 MPa, 9 MPa, and 10 MPa are selected within the scope of the aeration pressure. Run the simulation models. Figures 11 and 12 present the volume and pressure changing of the accumulator. The rotational speed of the hydraulic motor is shown in Figure 13. The SOC of the battery is shown in Figure 14.

Based on the figures above, it can be seen that the pressure of the accumulator is proportional to its aeration pressure. When the boom cylinder piston is expanding in the second working cycle, the hydraulic motor is driven by the oil stored in the accumulator. The volume changing of the pressure oil in the accumulator increases along with the increasing of the aeration pressure. It leads to increasing the flow of the pressure oil in the energy recovery system. The pressure difference between the inlet and outlet of the motor is increasing as well. Because of the constant displacement hydraulic motor, the flow rate through the hydraulic motor is proportional to its rotational speed. The output torque of the motor is increasing gradually based on the pressure difference. The generator is connected to the hydraulic motor coaxially. According to the characteristics of the generator, the output torque of the motor is increasing as well. Hence the electric energy produced by the generator and SOC of the battery are increasing.

#### 5.1.2. The Initial Volume

In order to analyze the influence of the different initial volume on the energy recovery efficiency, four values 450 L, 470 L, 480 L, and 500 L are selected within the scope of the initial volume under the condition that the aeration pressure and the highest working pressure of the accumulator are keeping in 10 MPa and 18 MPa.

**Figure 15:**
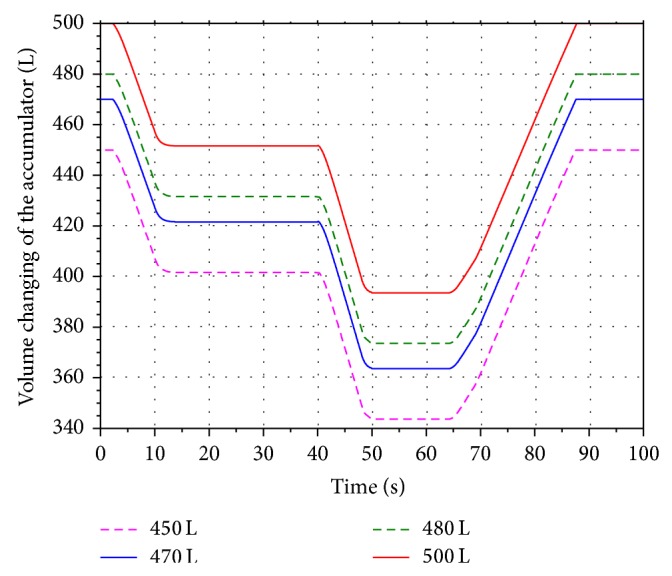
Volume changing of the accumulator.

**Figure 16:**
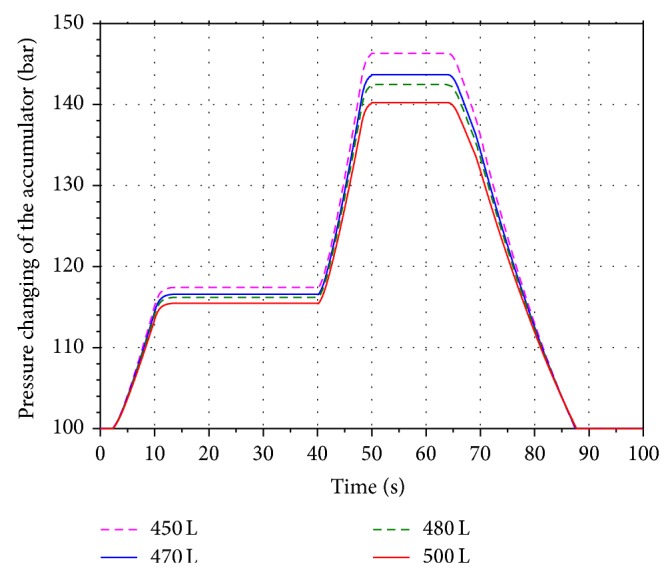
Pressure changing of the accumulator.

**Figure 17:**
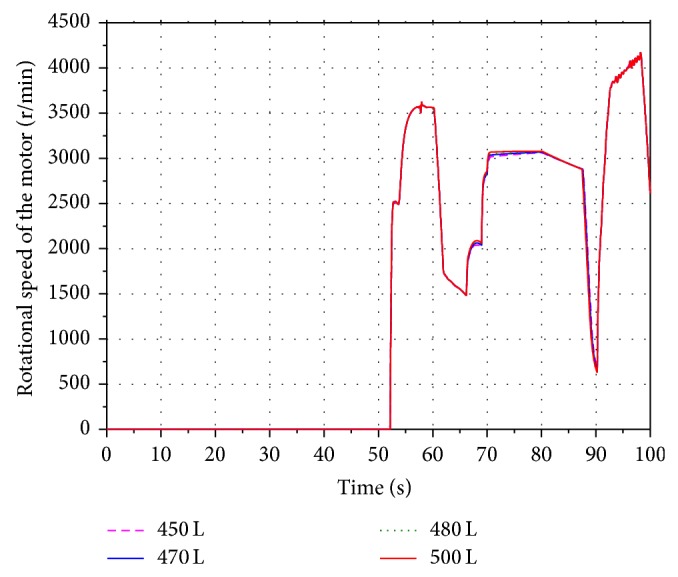
Rotational speed of the hydraulic motor.

**Figure 18:**
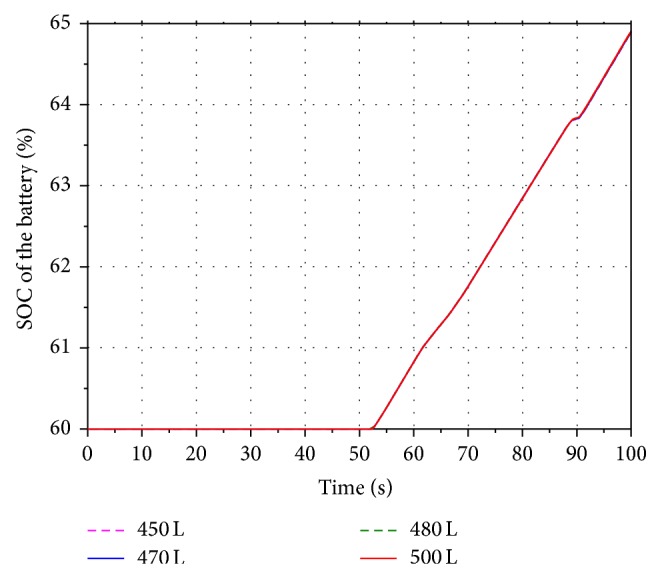
SOC of the battery.

Figures 15 and 16 show the volume and pressure changing of the accumulator. Keeping the aeration pressure unchanged, the simulation results indicate that the pressure of the accumulator is inversely proportional to the initial volume. The corresponding rotational speeds of the hydraulic motor are shown in Figure 17. The SOC of the battery is shown in Figure 18. It can be seen that the value of SOC is not changing with the different initial volume. In other words, the value of the initial volume does not have an effect on the improving of the boom energy recovery efficiency.

### 5.2. Analysis of the Hydraulic Motor

The hydraulic motor is used to drive the generator in the boom energy recovery system. The energy recovery system is determined by the performance of the motor. So it is essential to do some research on the hydraulic motor. The displacement is the most important parameter of the hydraulic motor. The simulations of the displacement and the type of the motor are shown below.

#### 5.2.1. The Displacement

In order to analyze the influence of the different displacement on the energy recovery efficiency, four values 60 mL/r, 80 mL/r, 100 mL/r, and 120 mL/r are selected as the displacement of the motor.

**Figure 19:**
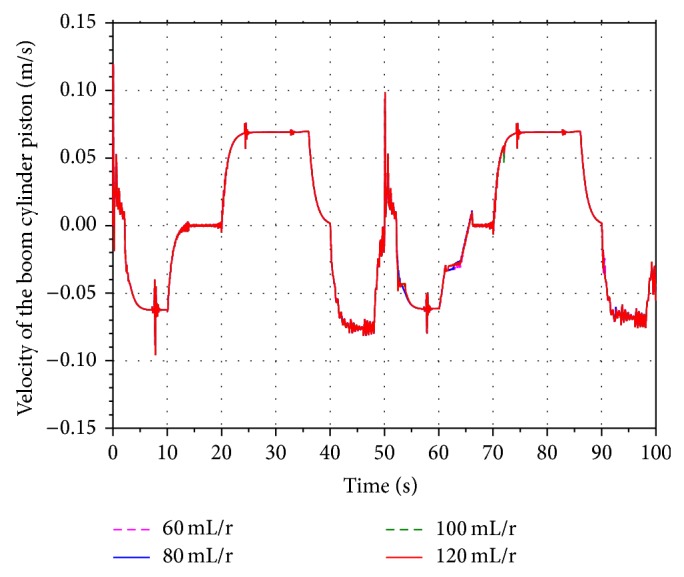
Velocity of the boom cylinder piston.

Figure 19 shows the velocity of the boom cylinder piston in the hybrid hydraulic excavator. Based on the working process of the hydraulic excavator, the boom cylinder is contracting during 52 s–69 s and 90 s–100 s. The hydraulic motor is driven by the pressure oil flowing into the return oil line. The result shows that the contracting velocity of the cylinder decreases along with the decreasing of the displacement. However, it does not have an effect on the normal work of the hydraulic excavator.

**Figure 20:**
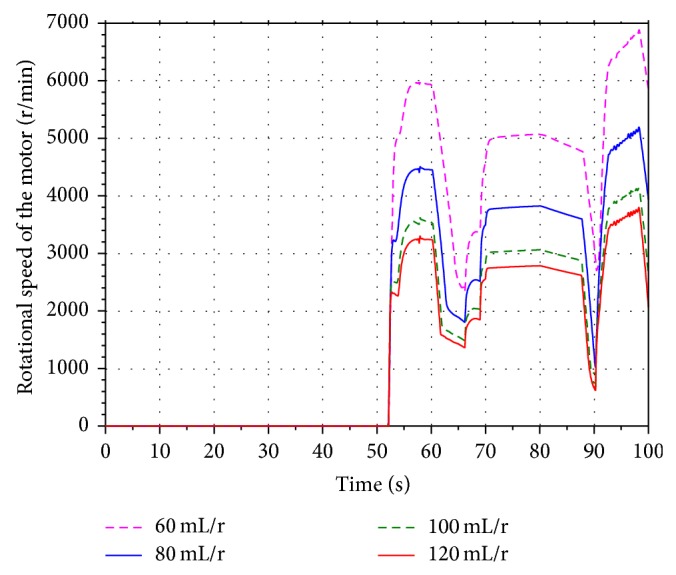
Rotational speed of the hydraulic motor.

**Figure 21:**
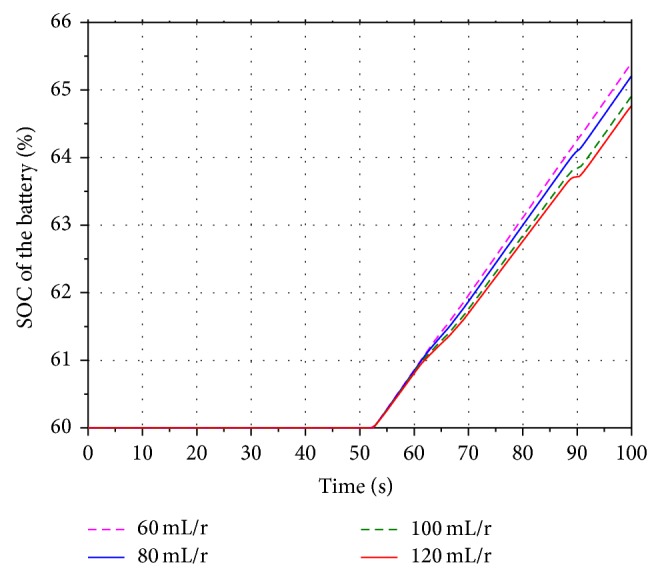
SOC of the battery.

Figures 20 and 21 show the rotational speed of the hydraulic motor and SOC of the battery with the different displacement. According to the characteristics of the hydraulic motor, the rotational speed of the motor is inversely proportional to the displacement. When the displacement is 60 mL/r, the SOC of the battery reaches the maximum value. It indicates that the SOC of the battery increases along with the decreasing of the displacement.

#### 5.2.2. The Type of the Hydraulic Motor

Because of the complex working condition of the hydraulic excavator, the velocity of the boom cylinder piston ranges from 0 to 0.1 m/s. The flow rate of the pressure in the return oil line ranges large. So it is very important to select the hydraulic motor for improving the energy recovery system.

**Figure 22:**
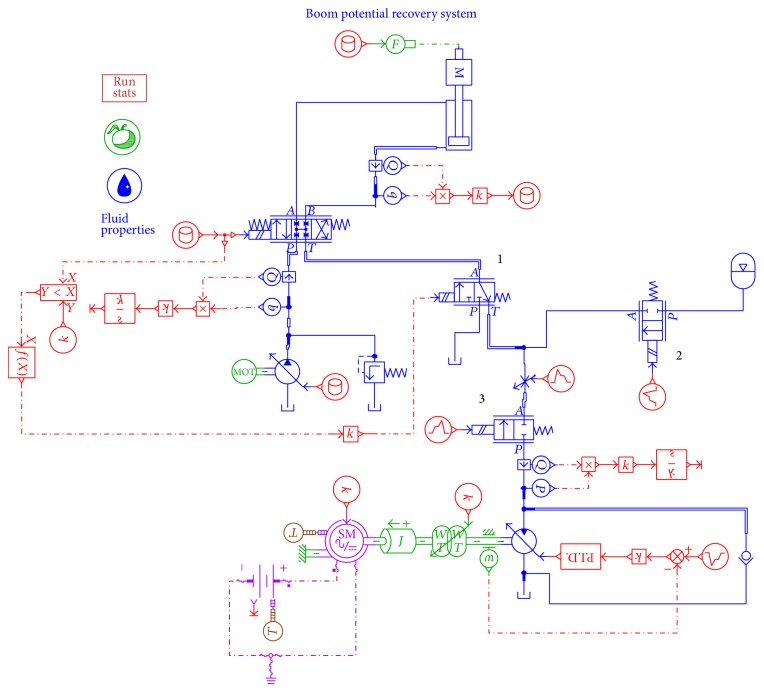
AMESim model with the variable displacement motor.

The hydraulic motor is divided into the constant displacement and variable displacement motor. The AMESim model with the variable displacement motor is shown in Figure 22.

**Figure 23:**
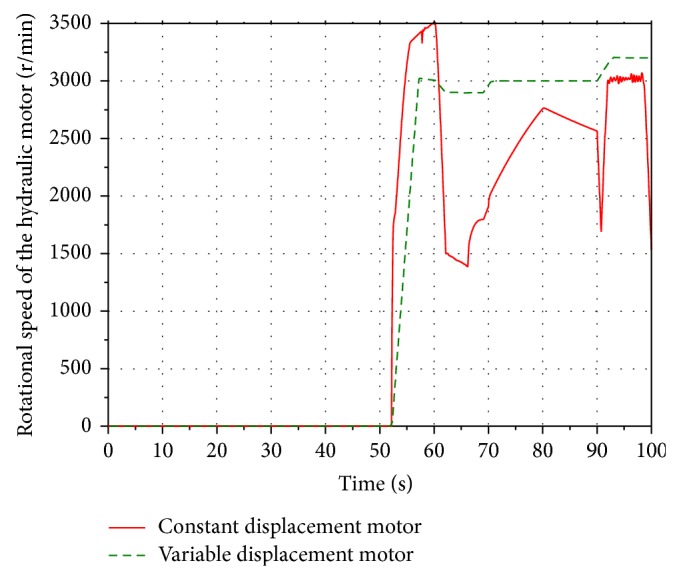
Rotational speed of the hydraulic motor.

**Figure 24:**
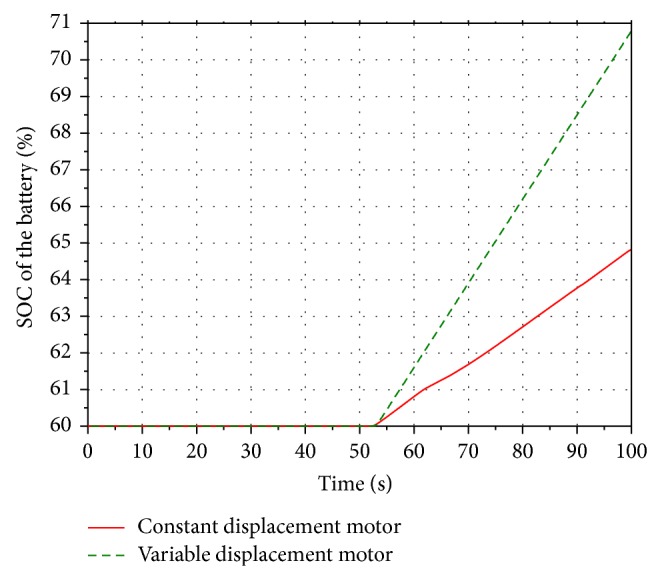
SOC of the battery.

Run the simulation. Compared with the models with the constant displacement motor shown in Figure 6, the rotational speed of the hydraulic motor in two simulation models is presented in the Figure 23.  Figure 24 shows the SOC range of the battery.

It can be seen that the rotational speed of the constant displacement motor is ranging between 1300 r/min and 3500 r/min during a working cycle, while the rotational speed of the variable displacement motor remains at 3000 r/min. The SOC of the battery of the constant displacement motor system increases from 60% to 64.8%, while the SOC of the battery of the variable displacement motor system reaches 70.8%. Compared with the constant displacement motor system, more boom potential energy of the energy recovery system with the variable displacement motor is recovered and stored in the battery. Hence, the energy recovery efficiency of the variable displacement motor system is higher than the system with the constant displacement motor.

## 6. Conclusions

1. Based on the simulation of the working devices in the conventional hydraulic excavator, the energy which can be recovered of the three cylinders is calculated. Taking the complexity and cost of the system into consideration, this paper proposed a novel boom potential energy recovery system for the parallel hybrid excavator. The boom energy regeneration unit consists of an accumulator, a hydraulic motor, an electric generator, and a battery. Compared with the conventional energy recovery system, the proposed system makes sure that the generator can rotate continuously in a high speed during a working cycle. The AMESim models of the two boom energy recovery systems are built and the results show that the proposed energy recovery system brings higher energy recovery efficiency than the conventional energy recovery system.
2. The mathematical models of the main components including boom cylinder, hydraulic motor, and hydraulic accumulator are built. The natural frequency of the proposed energy recovery system is calculated based on the mathematical models. In order to improve the natural frequency and response speed of the system, some measures should be taken based on the expression of the natural frequency, such as reducing the loop oil volume *V*, the leakage coefficients of the energy recovery system *C*, and the total moment of inertia of the hydraulic motor *J*.
3. The influence of the main components including hydraulic motor and hydraulic accumulator on the energy recovery efficiency of the proposed system is analyzed. The key parameters of the accumulator include the aeration pressure and the initial volume. The energy recovery efficiency of the proposed system can be improving to some extent by increasing the aeration pressure, while changing of the initial volume does not have an effect on improving of the energy recovery efficiency.

The hydraulic motor is used to drive the generator in the boom energy recovery system. The displacement is the most important parameter of the hydraulic motor. The energy recovery efficiency can be improving on the premise of normal working by decreasing the displacement of the motor. Since the flow rate of the pressure in the return oil line ranges large, the generator can rotate continuously in a high speed by selecting the variable displacement motor in the return oil line. In order to improve the energy efficiency, according to the characteristics of the generator, the variable displacement hydraulic motor should be chosen in the return oil line.

## Figures and Tables

**Table 1:** Input energy and excess energy of the three cylinder systems.

	Input energy *E*_in_ (J)	Excess energy *E*_ex_ (J)	Percentage *E*_ex_/*E*_in_ (%)
Boom system	1.26399*E*6	0.598651*E*6	47.36
Bucket rod system	0.67635*E*6	62118	9.18
Bucket system	0.74053*E*6	52944.6	7.15

**Table 2:** Setting parameters for the two AMESim models.

Common parts	Parameters	Values
Boom cylinder	Piston diameter (mm)	350
	Rod diameter (mm)	220
	Length of stroke (m)	1.8

Generator	Reference voltage (V)	50

Battery	Nominal capacity (Ah)	50
	State of charge (%)	60

**Table 3:** Input energy and the energy stored in the battery.

System	Input energy *E*_in_ (J)	Energy stored in the battery *E*_st_ (J)	Percentage *E*_st_/*E*_in_ (%)
Conventional system	7.6460*E*6	0.504*E*6	6.6%
Proposed system	6.8923*E*6	1.008*E*6	14.6%
